# Knowledge, attitudes, and practices of the general population of Pakistan regarding typhoid conjugate vaccine: findings of a cross-sectional study

**DOI:** 10.3389/fpubh.2023.1151936

**Published:** 2023-06-02

**Authors:** Muhammad Junaid Tahir, Musharaf Zaman, Junaid Saffi, Muhammad Sohaib Asghar, Waleed Tariq, Faizan Ahmed, Rabia Islam, Usman Shakeel Farooqui, Irfan Ullah, Muhammad Saqlain, Kaleem Ullah, Ali Ahmed

**Affiliations:** ^1^Department of Medicine, Lahore General Hospital, Lahore, Pakistan; ^2^Department of Anesthesia, Pakistan Red Crescent Teaching Hospital, Lahore, Pakistan; ^3^Department of Internal Medicine, Dow University of Health Sciences-Ojha Campus, Karachi, Pakistan; ^4^Department of Medicine, Punjab Medical College, Faisalabad, Pakistan; ^5^Department of Internal Medicine, Dow Medical College, Karachi, Pakistan; ^6^Department of Medicine, Kabir Medical College, Gandhara University, Peshawar, Pakistan; ^7^Department of Pharmacy, Quaid-I-Azam University, Islamabad, Pakistan; ^8^Department of Hepatobiliary Surgery and Transplant, Pir Abdul Qadir Shah Jeelani Institute of Medical Sciences, Gambat, Pakistan; ^9^Department of Pharmacy, School of Pharmacy, Monash University, Subang Jaya, Selangor, Malaysia

**Keywords:** typhoid fever, enteric fever, extended drug resistance, willingness, booster dose

## Abstract

Typhoid fever, a common enteric disease in Pakistan, caused by *Salmonella typhi*, is becoming an extended drug-resistant organism and is preventable through the typhoid conjugate vaccine (TCV). Public adherence to preventive measures is influenced by knowledge and attitude toward the vaccine. This study investigates the knowledge, attitudes, and practices of the general population of Pakistan toward TCV. The differences in mean scores and factors associated with typhoid conjugate vaccine knowledge, attitudes, and practices were investigated. A total of 918 responses were received with a mean age of 25.9 ± 9.6, 51% were women, and 59.6% had graduation-level education. The majority of them responded that vaccines prevent illness (85.3%) and decrease mortality and disability (92.6%), and typhoid could be prevented by vaccination (86.7%). In total, 77.7 and 80.8% considered TCV safe and effective, respectively. Of 389 participants with children, 53.47% had vaccinated children, according to the extended program on immunization (EPI). Higher family income has a higher odds ratio (OR) for willingness toward booster dose of TCV [crude odds ratio (COR) = 4.920, *p*–value <0.01; adjusted odds ratio (aOR) = 2.853, *value of p* <0.001], and negative attitude regarding the protective effect of TCV has less willingness toward the booster dose with statistical significance (COR = 0.388, *value of p* = 0.017; aOR = 0.198, *value of p* = 0.011). The general population of Pakistan had a good level of knowledge about the benefits of TCV, and attitude and practices are in favor of the usage of TCV. However, a few religious misconceptions are prevalent in public requiring the efforts to overcome them to promote the usage of vaccines to prevent the disease and antibiotic resistance.

## Introduction

Typhoid fever is a systemic infection characterized by fever, vomiting, and diarrhea caused by *Salmonella typhi* (*S. typhi*) (1, 2). The disease is transmitted by consuming fecal-contaminated water and food (3). Annually, typhoid affects 11 to 20 million people with 128,000 to 161,000 deaths worldwide.^4^ Underprivileged communities and children are at higher risk for typhoid fever (4). Children younger than 5 years of age had the highest rates of morbidity and mortality (12.6% of cases and 17% of deaths), followed by children aged 5 to 9 years (56% of cases and 59% of deaths). Typhoid fever is also a common disease in Pakistan, affecting 451.7 people per 100,000 each year (5). The improvements in sanitary infrastructure significantly control enteric fever in developed countries. The misusage of antibiotics in lower-middle-income countries (LMICs) for the treatment of *S. typhi* is growing antibiotic resistance, raising danger to global public health (1, 6). The mortality rate due to *S. typhi* had also increased, attributing to antibiotic resistance. *S. typhi* strains resistant to antibiotics such as chloramphenicol, ampicillin, trimethoprim–sulfamethoxazole, fluoroquinolones, and third-generation cephalosporin are labeled as extended drug-resistant (XDR) (7).

Pakistan had one of the highest rates of typhoid cases in South Asia. The rising level of antibiotic resistance in the country is raising global fear. The dire state of the sewage and water system along with low vaccination rates, non-compliance to treatment, and overcrowding are the major elements contributing to the spread of XDR typhoid within Pakistan (8). Furthermore, in 2016, XDR typhoid outbreak occurred in Sindh, Pakistan (9). The prevalence of positive cultures of *S. paratyphi* A raised from 0.2% in 2017 to 0.3% in 2018, then up to 0.9% in 2019 and increasing the prevalence of XDR typhoid fever across the country, indicating spread outside Sindh (9). Without active and effective measures, the preexisting and increased burden of XDR typhoid is expected to worsen, given the strain coronavirus disease 2019 (COVID-19) is putting on the healthcare system (10, 11). The prevalence of diseases, disabilities, and deaths has been significantly reduced because of vaccination (12).

Vaccines prevent 4–5 million people from dying from fatal diseases each year (13). Numerous success stories against polio, tetanus, influenza, hepatitis B, diphtheria, pertussis, measles, mumps, and rubella (MMR) had been made possible by effective vaccination. Typhoid vaccination had been proven effective for the prevention and control of typhoid. Pakistan is the first country to introduce typhoid conjugate vaccine (TCV) in the extended program on immunization (EPI) and had vaccinated over 30 million children in the country with an effectiveness of 95% against protection from typhoid (14). The first single dose of TCV provides prolonged immunity and is effective and safe for children between 6 months and 2 years of age (15).

**Figure 1:**
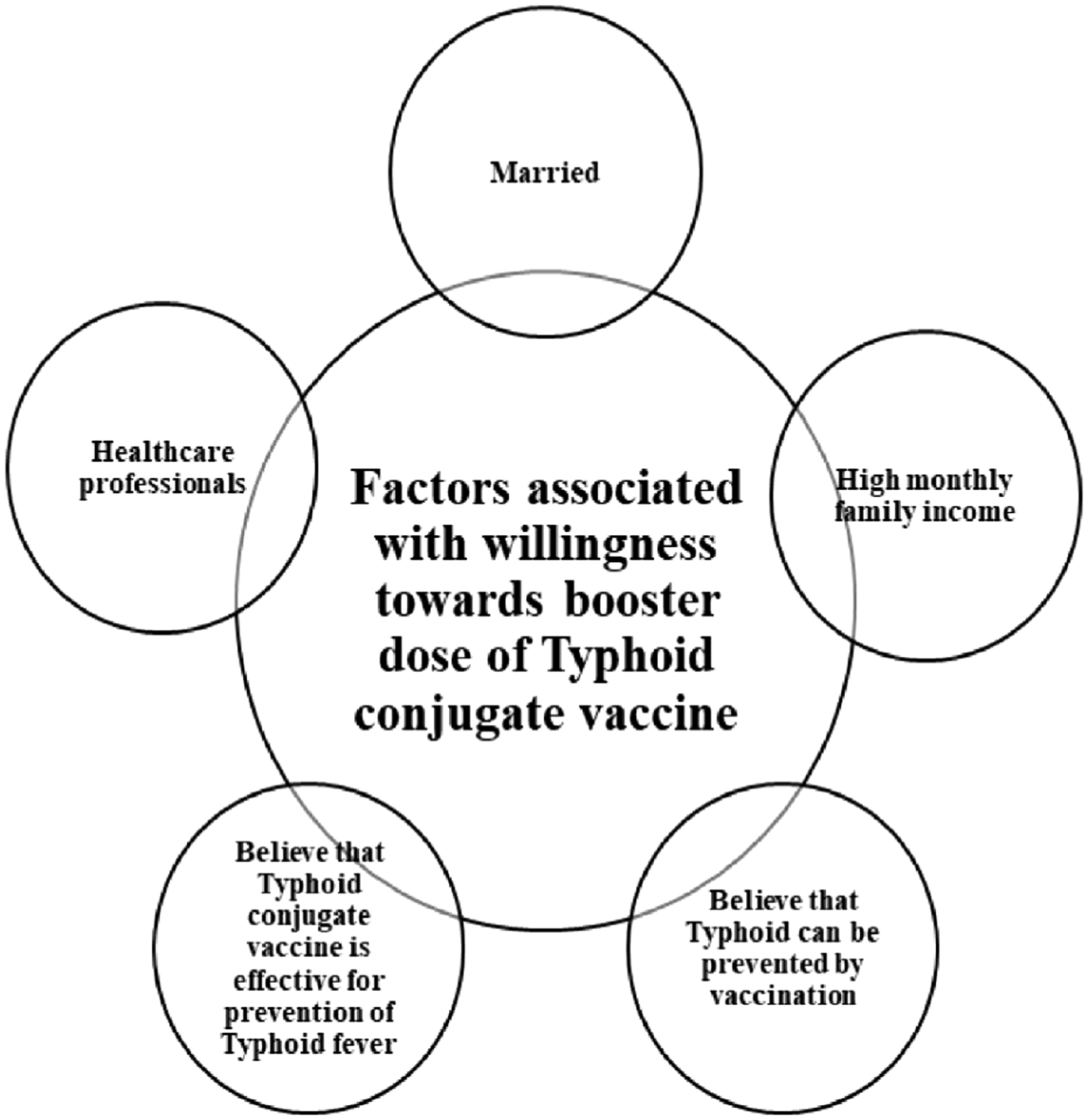
Factors associated with willingness toward booster dose of typhoid conjugate vaccine.

**Figure 2:**
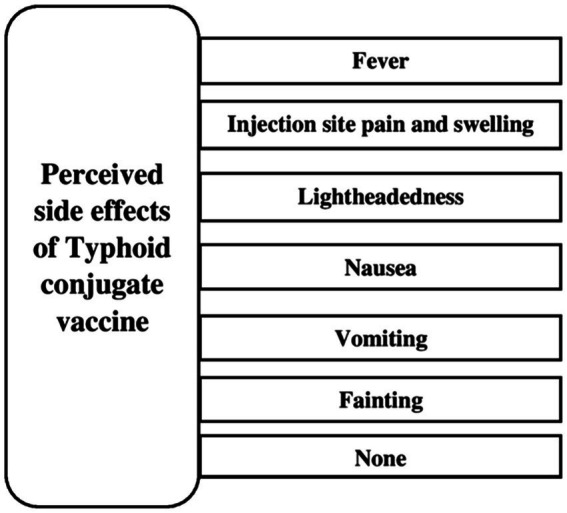
Perceived side effects of typhoid conjugate vaccine.

**Figure 3:**
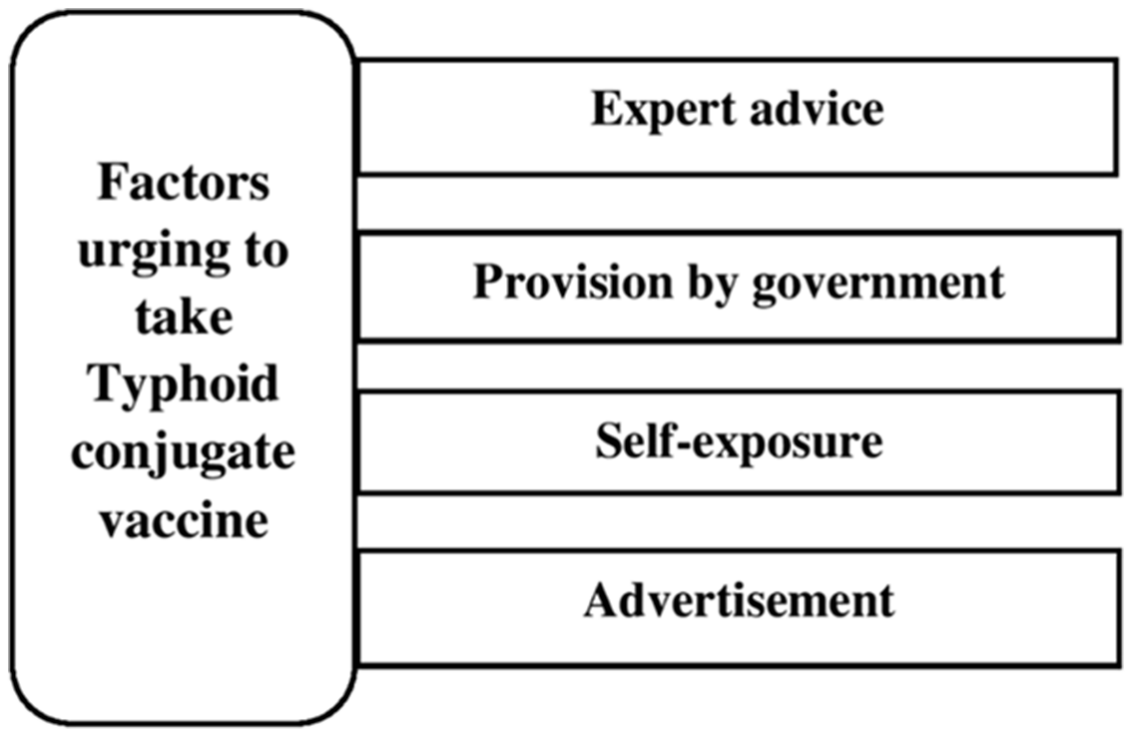
Factors urging to take typhoid conjugate vaccine.

The vaccine coverage gap does, however, exist between nations and even within a nation (16). The World Health Organization (WHO) identified vaccine reluctance as one of the threats to global health (17). Vaccine hesitation had been caused by several factors, including erroneous safety worries about immunization as a result of measles, diphtheria, and pertussis outbreaks (12). Influential religious and political figures and false religious beliefs greatly influenced vaccine refusal (18, 19). Personal or philosophical convictions, such as the idea that a healthy lifestyle will prevent sickness or that active immunity obtained after natural infection is superior to that acquired by vaccination, are other factors contributing to vaccination refusal (20) (Figures 1–3).

The vaccination rates of Pakistan’s population are below the required levels worldwide (21). The vaccination rates for Bacillus Calmette–Guérin (BCG), polio combined with DPT (Diptheria, Pertussis, and Tetanus), and measles are each stated to be at 80, 65, and 67%, respectively (21). This is a result of logistical challenges, inadequately qualified medical staff, inadequate parental education and awareness, and political, religious, and commercial influences on the promotion of vaccine products (21–23). Education and vaccines are the most powerful tools, proven to be valuable, against preventable diseases (24, 25). Previously, in Pakistan, the literature had been published regarding awareness and attitudes toward TCV involving a specific and localized population (26). We are conducting this study among the general population of Pakistan to assess the knowledge, attitude, and practices regarding TCV and willingness toward the booster dose of TCV.

## Materials and methods

### Study design and study setting

A cross-sectional, web-based study was conducted among the general population of Pakistan, involving participants from all provinces, from 15 October 2021 to 30 November 2021.

### Ethical considerations

Participation in this study was completely voluntary. Before beginning the survey, each participant provided informed consent and was given the option to withdraw from the study at any time before submitting their response. The anonymity of the participants was assured by not collecting any personal information such as name, email, or any other contact information. The review committee of Pir Abdul Qadir Shah Jeelani Institute of Medical Sciences, Gambat, Pakistan, with reference number: IRB/22/13, approved ethical approval for the study. Furthermore, the STROBE checklist is followed to report this study.

### Sample size

The population of Pakistan is 227.3 million with a median age of 23.2 years and 51.5 and 48.5% being men and women, respectively, with the literacy rate of 62.3% (27, 28). The online Rao soft calculator [*n = ^Nx^I* ((*N*–1)*E^2^* + *x*)] was used to calculate the sample size of the study (29). A minimum sample size of 846 was calculated with a 98% confidence interval, 50% proportion of the population, and 4% margin of error. A total of 918 responses were included with a 96% response rate.

### Data collection

A Google^®^ form was used to collect the responses through a convenient sampling method. The questionnaire link was circulated among friends and contacts in multiple social media groups such as WhatsApp and Facebook. Individuals were unable to understand the language of the survey, and non-Pakistani nationals were excluded from the study.

### Questionnaire designing

The questionnaire was designed after a substantial literature review (12, 16, 24, 30, 31). It was validated by medical education and public health experts. To assess the relevancy and simplicity of the questionnaire, a pilot study was conducted with 40 individuals from the target population. The questionnaire was comprised of demographic information and questions about knowledge, attitude, and practices regarding TCV. The sociodemographic characteristics include age, gender, education, occupation, residence, marital and employment status, monthly family income, and the number of household individuals. The knowledge section was comprised of action, benefits, availability, cost, source of information, side effects of TCV, and awareness about the TCV vaccination program conducted by the Government of Pakistan. The attitude assessed participants’ beliefs about safety, efficacy, recommendation, and need for TCV for children. Furthermore, attitudes regarding religious beliefs toward vaccines were also inquired. Regarding practices of TCV, vaccination of own child, to comply with the booster dose, and family restrictions regarding vaccine were inquired.

### Statistical analysis

The statistical package for social sciences (SPSS) version 21 (IBM) was used for data analysis. Mean and standard deviation were used to present the numerical variables, and for categorical data, frequencies and percentages were used. Depending upon the nature of the variables, inferential analysis was applied. Univariate and multivariate logistic regression analyses of sociodemographic, knowledge, and attitude with willingness for a booster dose of TCV were conducted, and a *value of p* of <0.05 was considered significant.

## Results

### Sample characteristics

A total of 918 responses were received with a response rate of 96%. The mean age and number of family members were 25.9 ± 9.6 and 5.9 ± 3.7, respectively. Regarding sociodemographic information, 51% (468) were women, 25.7% (236) were married, and 39.5% (363) were employed. Among the majority of the people, 82.7% (759) belonged to urban areas, 59.6% (547) had graduation-level education, and 60.2% (533) had a monthly family income of more than 50,000 PKR (Table 1).

**Table 1:** General characteristics and demographics of the study participants (*n* = 918).

Variables	Characteristics	Frequency/percentage
Age (in years)	Mean (SD)	25.9 (9.6)
Gender	Female	468 (51.0%)
	Male	450 (49.0%)
Marital status	Unmarried	682 (74.3%)
	Married	236 (25.7%)
Residence	Rural	159 (17.3%)
	Urban	759 (82.7%)
Monthly family income (PKR)	< 25,000	162 (17.6%)
	25,000–50,000	203 (22.1%)
	> 50,000	553 (60.2%)
Level of education	No formal education	25 (2.7%)
	Primary up to Matric	68 (7.4%)
	Secondary/Intermediate	191 (20.8%)
	Graduate	547 (59.6%)
	Post-graduation	87 (9.5%)
Number of household members	Mean (SD)	5.9 (3.7)
Employment status	Unemployed	555 (60.5%)
	Employed	363 (39.5%)
Occupation	Healthcare workers	253 (27.6%)
	Others/No occupation	665 (72.4%)

PKR, Pakistani Rupee; SD, standard deviation.

### Assessment of knowledge in the participants

The majority of the people (783) responded that vaccine is used to prevent illness (85.3%), 834 (90.8%) reported that healthy child needs vaccination, 850 (92.6%) reported that vaccine lowers disability and mortality, and 796 (86.7%) reported that typhoid could be prevented by vaccination. In total, 54.2% (498) were aware of TCV, and 52.1% (478) of TCV vaccination programs conducted by the Government of Pakistan. Mass media [35.1% (322)] and healthcare workers [16.2% (149)] were major sources of information. Regarding side effects, fever [462 (50.3%)] and injection site pain and swelling [453 (49.3%)] were considered the most common reactions to TCV (Table 2).

**Table 2:** Knowledge about typhoid as a disease and vaccination of the study participants (*n* = 918).

Variables	Characteristics	Frequency (%)
What do you think vaccination do?	I do not know	45 (4.9%)
	It’s just an injection	22 (2.4%)
	It’s used to cure disease	68 (7.4%)
	Prevent illness	783 (85.3%)
Do you know that even healthy child needs	No	84 (9.2%)
vaccination?	Yes	834 (90.8%)
Do you know vaccination decreases the rates of mortality and disabilities?	No	68 (7.4%)
	Yes	850 (92.6%)
Do you know typhoid could be prevented by	No	122 (13.3%)
vaccination?	Yes	796 (86.7%)
Do you know about Typhoid Conjugate	No	420 (45.8%)
Vaccine?	Yes	498 (54.2%)
Do you know that the government of Pakistan had started typhoid vaccination in different districts of Pakistan?	No	440 (47.9%)
	Yes	478 (52.1%)
How did you come to know about typhoid vaccination?	Doctors and Nurses	149 (16.2%)
	Friends And Relatives	139 (15.1%)
	Mass Media (TV/Newspaper)	322 (35.1%)
	Personal knowledge	126 (13.7%)
	I do not know	182 (19.8%)
Do you know which age group children should get typhoid vaccination?	0–5 years	335 (36.5%)
	5–13 years	246 (26.8%)
	More than 13 years	98 (10.7%)
	I do not know	239 (26.0%)
What do you know about booster dose?	Given to cover missed dose	71 (7.7%)
	Gives more protection	667 (72.7%)
	I do not know	180 (19.6%)
Is typhoid vaccine available free in Pakistan or patient bear its price?	Patient bear its price	208 (22.7%)
	Typhoid vaccine available free in Pakistan	498 (54.2%)
	I do not know	212 (23.1%)
What side effects does TCV have?	Fever	462 (50.3%)
	Lightheadedness	187 (20.4%)
	Nausea	173 (18.8%)
	Vomiting	102 (11.1%)
	Fainting	41 (4.5%)
	Pain and swelling at injection site	453 (49.3%)
	None	35 (3.8%)
	I do not know	478 (52.1%)

TV, Television; TCV, Typhoid Conjugate Vaccine.

### Assessment of the attitude of participants

In total, 77.7% (713) and 80.8% (742) considered TCV safe and effective, respectively, and 91.8% (843) were in favor of vaccination programs designed by healthcare authorities. In total, 92.3% (847) would advise TCV to family and friends, 617 (67.2%) would strongly recommend others to vaccinate their children, and 53.3% (489) followed expert advice for TCV vaccination. Only 21.6% (198) had fear about TCV, and 13.1% (120) considered it a conspiracy theory against Pakistan; however, 623 (67.9%) believed that it may contain contents forbidden by Islamic law (Table 3).

**Table 3:** Attitude of the study participants toward typhoid vaccination (*n* = 918).

Variables	Characteristics	Frequency (%)
Are you in favor of obligatory vaccination	No	75 (8.2%)
programs designed by the health authorities?	Yes	843 (91.8%)
Will you advice your relatives and family	No	71 (7.7%)
members to immunize their children?	Yes	847 (92.3%)
Do you believe that typhoid conjugate vaccine is safe?	No	36 (3.9%)
	Yes	713 (77.7%)
	I do not know	169 (18.4%)
Do you believe that typhoid conjugate vaccine is effective for prevention of typhoid fever?	No	34 (3.7%)
	Yes	742 (80.8%)
	I do not know	142 (15.5%)
Will you recommend vaccine to other children?	Discourage taking vaccine	31 (3.4%)
	Only if they ask	142 (15.5%)
	Strongly recommend	617 (67.2%)
	No comments	128 (13.9%)
Will you vaccinate the child if he/she is already sick?	No	536 (58.4%)
	Yes	382 (41.6%)
Do you have any fears about this vaccination?	No	720 (78.4%)
	Yes	198 (21.6%)
What encourages you to get the child vaccinated with Typhoid Conjugate Vaccine?	Advertisements	28 (3.1%)
	Expert Advice	489 (53.3%)
	Government Provides Vaccination	274 (29.8%)
	Self-experience	98 (10.7%)
	Will not vaccinate my child	29 (3.2%)
Would you support it if it is added in Govt. vaccination program?	No	62 (6.8%)
	Yes	856 (93.2%)
Do you think it is an international conspiracy against Pakistani children?	No	798 (86.9%)
	Yes	120 (13.1%)
Do you think it may contain contents forbidden by Islamic law?	No	199 (21.7%)
	Yes	623 (67.9%)
	I do not know	96 (10.5%)

### Assessment of practices of participants

Only 389 (42%) participants had children, and 208 (22.7%) had vaccinated their children according to EPI including TCV. In total, 763 (83.1%) would comply with the booster dose, and 829 (90.3%) had family restrictions regarding vaccination. The majority would consult with the doctor for side effects of vaccination [533 (58.1%)], the missed dose of TCV [842 (91.7%)], and the next vaccination [845 (92.0%)] (Table 4).

**Table 4:** Practice of study participants toward typhoid vaccination (*n* = 918).

Variables	Characteristics	Frequency
Have you given your children the obligatory vaccines including typhoid vaccine?	No	69 (7.5%)
	Yes	208 (22.7%)
	Given vaccines other than typhoid	112 (12.2%)
	I have no children	529 (57.6%)
Was the vaccine from Government Campaign or taken privately?	Government Campaign	262 (28.5%)
	Privately	87 (9.5%)
	Not vaccinated	34 (3.7%)
	Not applicable	535 (58.3%)
Would you comply with a booster dose?	No	155 (16.9%)
	Yes	763 (83.1%)
Do you have family restrictions regarding this vaccine?	No	89 (9.7%)
	Yes	829 (90.3%)
What side effects you think it has?*	Fever	462 (50.3%)
	Lightheadedness	187 (20.4%)
	Nausea	173 (18.8%)
	Vomiting	102 (11.1%)
	Fainting	41 (4.5%)
	Pain and swelling at injection site	453 (49.3%)
	None	35 (3.8%)
	I do not know	478 (52.1%)
What would you do if the child experience any of the side effect following vaccination?	Believe that vaccination is working	115 (12.5%)
	Consult a doctor	533 (58.1%)
	Give fever medication at home	197 (21.5%)
	Stop giving the vaccination	26 (2.8%)
	I do not know	187 (20.4%)
Who would you contact if the child missed the dose?	Doctor	842 (91.7%)
	Nurse	16 (1.7%)
	Pharmacist	21 (2.3%)
	Family or friends	14 (1.5%)
	Handle myself	25 (2.7%)
Who will you contact for next vaccination?	Doctor	845 (92.0%)
	Nurse	23 (2.5%)
	Pharmacist	28 (3.1%)
	Family or friends	22 (2.4%)

^*^This question had multiple options to choose with.

### Regression analysis of factors associated with willingness for a booster dose of typhoid vaccine

Being married has a significant association with willingness for a booster dose of TCV [crude odds ratio (COR) = 0.285, *value of p* < 0.001; adjusted odds ratio (aOR) = 0.562, *value of p* =0.038]. Participants with a monthly family income of more than 50,000 PKR have higher OR than those with lower income (COR = 4.920, *value of p* <0.01; aOR = 2.853, *value of p* < 0.001). Based on univariate and multivariate analyses, a negative attitude regarding the protective effect of TCV has less willingness toward the booster dose with statistical significance (COR = 0.388, *value of p* = 0.017; aOR = 0.198, *value of p* = 0.011) (Table 5).

**Table 5:** Regression analysis of factors associated with willingness for booster dose of typhoid vaccine.

Variables	Univariate	*p*-value	Multivariate	*p*-value
Gender				
Male	1.000	-	1.000	–
Female	1.652 (1.163–2.346)	0.005*	1.132 (0.735–1.744)	0.573
Age groups				
≤20 years (*n* = 199)	3.658 (1.738–7.702)	0.001*	0.875 (0.307–2.497)	0.803
21–30 years (*n* = 574)	5.236 (2.624–10.448)	<0.001*	1.520 (0.606–3.815)	0.372
31–50 years (*n* = 107)	1.434 (0.672–3.063)	0.351	0.968 (0.402–2.331)	0.942
>50 years (*n* = 38)	1.000	-	1.000	-
Marital status				
Unmarried	1.000	-	1.000	-
Married	0.285 (0.199–0.409)	<0.001*	0.562 (0.326–0.969)	0.038*
Residence				
Urban	1.000	-	1.000	-
Rural	0.255 (0.173–0.376)	<0.001*	0.673 (0.403–1.124)	0.130
Monthly family income (PKR)				
< 25,000	1.000	-	1.000	-
25,000–50,000	1.090 (0.694–1.710)	0.709	0.826 (0.496–1.374)	0.461
> 50,000	4.920 (3.138–7.714)	<0.001*	2.853 (1.655–4.916)	<0.001*
Number of household members				
1–2 (*n* = 56)	2.036 (0.874–4.740)	0.099	2.133 (0.850–5.354)	0.107
3–4 (*n* = 195)	1.890 (1.138–3.140)	0.014*	1.414 (0.795–2.516)	0.239
5–6 (*n* = 414)	1.561 (1.050–2.321)	0.028*	1.066 (0.675–1.684)	0.784
>6 (*n* = 253)	1.000	-	1.000	-
Level of Education				
No formal education	0.250 (0.099–0.634)	0.003*	0.692 (0.221–2.165)	0.526
Primary up to Matric	0.514 (0.257–1.028)	0.060	1.382 (0.618–3.093)	0.431
Secondary/Intermediate	2.214 (1.154–4.247)	0.017*	2.013 (0.942–4.305)	0.071
Graduate	2.168 (1.249–3.763)	0.006*	1.370 (0.729–2.576)	0.328
Post-graduation	1.000	-	1.000	-
Employment status				
Employed	0.589 (0.416–0.833)	0.003*	1.024 (0.634–1.653)	0.923
Unemployed	1.000	-	1.000	-
Occupation				
Healthcare workers	3.635 (2.147–6.155)	<0.001*	2.339 (1.314–4.163)	0.004*
Others/No occupation	1.000	-	1.000	-
Do you know typhoid could be prevented by vaccination?				
No	0.146 (0.097–0.222)	<0.001*	0.337 (0.196–0.580)	<0.001*
Yes	1.000	-	1.000	-
Do you know that Government of Pakistan have started typhoid vaccine drive?				
No	0.505 (0.354–0.719)	<0.001*	0.667 (0.421–1.056)	0.084
Yes	1.000	-	1.000	-
Do you believe that typhoid conjugate vaccine is safe?				
No	0.790 (0.384–1.627)	0.523	1.595 (0.756–3.366)	0.267
Yes	6.707 (4.518–9.957)	<0.001*	2.078 (0.571–7.566)	0.220
I do not know	1.000	-	1.000	-
Do you believe that typhoid conjugate vaccine is effective for prevention of typhoid fever?				
No	0.388 (0.178–0.845)	0.017*	0.198 (0.057–0.688)	0.011*
Yes	6.417 (4.254–9.680)	<0.001*	1.379 (0.686–2.771)	0.367
I do not know	1.000	-	1.000	-
Do you have any fears about this vaccination?				
No	2.965 (2.045–4.299)	<0.001*	1.117 (0.672–1.856)	0.670
Yes	1.000	-	1.000	-
Do you think it is an international conspiracy against Pakistani children?				
No	1.000	-	1.000	-
Yes	0.389 (0.252–0.601)	<0.001*	0.979 (0.496–1.933)	0.952
Do you think it may contain contents forbidden by Islamic law?				
No	6.642 (4.437–9.943)	<0.001*	1.988 (1.060–3.728)	0.032*
Yes	1.469 (0.869–2.484)	0.151	0.968 (0.427–2.196)	0.939
I do not know	1.000	-	1.000	-

PKR, Pakistani Rupee. Significant *p*-values are denoted by *. The dependent variable compliance with a booster dose of typhoid vaccine has been responded to as ‘Yes’.

## Discussion

Typhoid conjugate vaccine (TCV) had been proven effective in reducing the burden of *S. typhi*-induced morbidity and mortality, especially in regions with XDR *S. typhi*. The routine TCV vaccination prevents antimicrobial resistance and misusage of antibiotics. This study assessed the knowledge, attitude, and practices of the general population of Pakistan regarding TCV. In total, 85.3% of our study population responded that vaccines prevent illnesses consistent with the study by Sankar et al. that 91.86% responded as vaccines provide prevention against illnesses (30). Only 54.2% of participants were aware of TCV being lower than the study by Hanif et al. as 75% of the sample population knew about TCV in Sindh, Pakistan (21). It can be attributed to the possibility that Hanif et al. had conducted the study in the vicinity of the vaccination center having the probability of raising consciousness and awareness about the vaccines.

Mass media (35.1%) and doctors and nurses (16.1%) followed by friends and relatives are the major sources of information for TCV while Debnath et al. had reported that healthcare workers (39.2%), mass media (25.6%), and relatives (12.8%) are the sources of knowledge for vaccines and vaccine-preventable diseases (31). Humans are now living in the era of mass and social media, and people depend on technological services even for basic daily needs making it the major source of information (23, 32). Social media campaigns had been proven effective in promoting awareness about health information such as their appeal, broad coverage, rapid provision, and cost-effectiveness (33).

The majority of the participants have a positive attitude toward TCV, as it was considered safe and effective by 77.7 and 80.8%, respectively. Memon et al. depicted that 75.7 and 87% believed that TCV is safe and effective, respectively (26). However, participants responded that TCV may contain contents forbidden by Islamic law (67.9%), have fear about the vaccine (21.6%), and is an international conspiracy against Pakistan (13.1%). Memon et al. reported that TCV is an international conspiracy against Pakistani children (73.5%), harmful (24.3%), and may have harmful contents (11.4%), and 92% would accept the vaccine if their issues and concerns were answered (26). Previously, religious leaders had spread the idea that polio vaccination will cause vaccine-induced infertility. Similarly, for the COVID-19 vaccine, people believed that it will make the population sterile, or the person getting vaccinated will die in 2 years (18). Similarly, the assumptions had been made against the other vaccines as TCV being comprised of harmful contents and would have hazardous effects on human health in the long run.

The advice from an expert (53.3%) and the provision of vaccination by the government (29.8%) would encourage the participants to vaccinate the children with TCV. Tahir et al. reported that the compulsion of vaccination by the government (48.06%) and the recommendation by the doctor (39.37%) are the reasons leading to vaccination against COVID-19 (12).

Healthcare workers are the most significant and trustworthy source of knowledge to the public for disease prevention and treatment, especially immunization, educating about the benefits of vaccination, and myths prevalent about the vaccine in society. Therefore, good communication between HCWs and the general population would promote vaccination and encourage them to vaccinate themselves and others in society (26). Majority, would consult a doctor for the missed dose of vaccine (58.1 %) and for side effects of vaccine (91.7%), and for the next vaccination (92.0%) corresponding with Sankar et al. as 70.2 and 29% would contact a doctor and nurse, respectively, for a missed dose of vaccine (30).

The higher income, education level, and less number of household members had more willingness for a booster dose of TCV. The higher income level contributes more to the affordability of medical treatments and utilizes available health facilities for healthy living and protection against diseases (34). Similarly, a higher education level raises awareness and knowledge regarding healthcare and reduces false beliefs resulting in a positive attitude toward the prevention and treatment of diseases and a willingness for vaccination. Older age, higher level of the perceived threat from the diseases, and positive attitude toward the vaccine are other factors contributing to the willingness for vaccination (35). The sociodemographic groups with low levels of education and income had lower willingness toward vaccination suggesting the modification in strategies to overcome the social inequalities to provide equal opportunities to vaccinate (36).

## Limitations of the study

This study, however, would have some limitations. First, convenient sampling was utilized for which there would be chances of selection bias. It was an online survey, and people with the facility to the Internet had only participated in the study. Second, this study would have recall bias. Third, compared to Pakistan’s overall population, our sample size was more educated, wealthier, and more urban. Finally, despite the fact that the replies were anonymous because the study was self-reported, participants may have felt under pressure to adopt the favorable attitudes and perceptions that were socially acceptable. This study calls for additional research focused on the population residing in rural areas and with low education levels.

## Conclusion

The general population of Pakistan had knowledge about the benefits of vaccination and the role of booster doses but was found to be lacking regarding the provision of TCV by the government in the country and its effectiveness against typhoid fever. The attitude and practices were reported to be in favor of promoting vaccination, but some false religious and national beliefs are also prevalent demanding the need to be addressed. People will also comply with the booster dose of TCV, indicating the awareness of the general population about the importance of completing a treatment course of vaccine for disease prevention. Furthermore, healthcare authorities, media, and government professionals should collaborate to conduct seminars and campaigns on domestic and national levels and should utilize electronic and print media to make an effort to address negative views about immunization and raise awareness about the disease, its adversities, mode of transmission, and the role of vaccines in the prevention of diseases and saving human lives.

## Data availability statement

The raw data supporting the conclusions of this article will be made available by the authors, without undue reservation.

## Ethics statement

The studies involving human participants were reviewed and approved by Pir Abdul Qadir Shah Jeelani Institute of Medical Sciences, Gambat. The patients/participants provided their written informed consent to participate in this study.

## Author contributions

IU, MT, and MS conceived the idea. MZ, JS, and UF collected the data. MA analyzed and interpreted the data. MZ, MT, JS, WT, FA, RI, UF, and AA performed write-up of the manuscript. AA, MA, MT, WT, KU, and MS reviewed the manuscript for intellectual content critically. All authors approved the final version of the manuscript.

## Conflict of interest

The authors declare that the research was conducted in the absence of any commercial or financial relationships that could be construed as a potential conflict of interest.

## Publisher’s note

All claims expressed in this article are solely those of the authors and do not necessarily represent those of their affiliated organizations, or those of the publisher, the editors and the reviewers. Any product that may be evaluated in this article, or claim that may be made by its manufacturer, is not guaranteed or endorsed by the publisher.
